# Exploring molecular mechanisms of drug resistance in bacteria and progressions in CRISPR/Cas9-based genome expurgation solutions

**DOI:** 10.1016/j.gmg.2025.100042

**Published:** 2025-02-16

**Authors:** K.E. Vivekanandan, P. Vinoth Kumar, R.C. Jaysree, T. Rajeshwari

**Affiliations:** a Department of Microbiology, PSG College of Arts and Science, Civil Aerodrome Post, Avinashi Road, Coimbatore, Tamil Nadu 641014, India; b Department of Microbiology, Shri Nehru Maha Vidyalaya, Shri Gambhirmal Bafna Nagar, Malumachampatti, Coimbatore 641050, India; c Department of Biotechnology, Nehru Arts and Science College, Thirumalayampalayam, Coimbatore 641105, India; d Department of Biotechnology, Dhanalakshmi Srinivasan College of Arts and Science for Women, Thuraiyur, Perambalur, Tamilnadu 621212, India

**Keywords:** Molecular mechanisms, Drug resistance, Bacteria, CRISPR/Cas9, Genome editing, Advancements

## Abstract

Antibiotic resistance in bacteria is a critical global health challenge, driven by molecular mechanisms such as genetic mutations, efflux pumps, enzymatic degradation of antibiotics, target site modifications, and biofilm formation. Horizontal gene transfer (HGT) further accelerates the spread of resistance genes across bacterial populations. These mechanisms contribute to the emergence of multidrug-resistant (MDR) strains, rendering conventional antibiotics ineffective. Recent advancements in CRISPR/Cas9-based genome editing offer innovative solutions to combat drug resistance. CRISPR/Cas9 enables precise targeting of resistance genes, facilitating their deletion or inactivation, and provides a potential method to eliminate resistance-carrying plasmids. Furthermore, phage-delivered CRISPR systems show promise in selectively killing resistant bacteria while leaving susceptible strains unaffected. Despite challenges such as efficient delivery, off-target effects, and potential bacterial resistance to CRISPR itself, ongoing research and technological innovations hold promise for using CRISPR-based antimicrobials to reverse bacterial drug resistance and develop more effective therapies. These abstract highlights the molecular mechanisms underlying bacterial drug resistance and explores how CRISPR/Cas9 technology could revolutionize treatment strategies against resistant pathogens.

## Graphical Abstract

**Figure:**
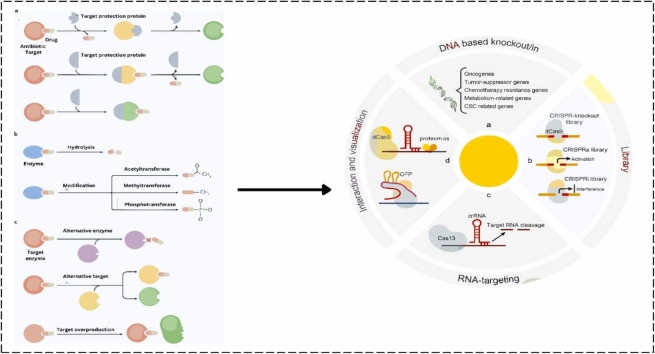

## Introduction

Antibiotic resistance has emerged as one of the most significant threats to global public health in the 21st century. The rapid spread of drug-resistant bacteria compromises the effectiveness of antibiotics, once considered the cornerstone of modern medicine. Bacterial pathogens that were previously treatable are now showing resistance to multiple classes of antibiotics, leading to increased mortality, prolonged hospital stays, and higher medical costs [1]. The World Health Organization (WHO) has labelled antibiotic resistance as a "global crisis," warning of the potential for a post-antibiotic era in which common infections and minor injuries may once again become fatal due to the lack of effective treatments. The molecular mechanisms by which bacteria acquire and disseminate drug resistance are diverse and highly efficient [2]. These mechanisms include spontaneous genetic mutations that alter the target sites of antibiotics, the activation of efflux pumps that expel antibiotics from bacterial cells, and the production of enzymes that degrade or inactivate the drugs. Bacteria also adopt physical strategies like biofilm formation, which encases bacterial communities in protective matrices, making it difficult for antibiotics to penetrate and act effectively [3]. However, one of the most powerful drivers of resistance is horizontal gene transfer (HGT), where bacteria exchange genetic material, including resistance genes, across species and strains. HGT facilitates the rapid spread of resistance traits, leading to the rise of multidrug-resistant (MDR) and even pan-resistant bacterial strains. In light of these challenges, traditional approaches to antimicrobial therapy are becoming increasingly inadequate, pushing researchers to explore innovative solutions. One such breakthrough is the CRISPR/Cas9 (Clustered Regularly Interspaced Short Palindromic Repeats/CRISPR-associated protein 9) genome editing system. Originally discovered as part of the bacterial immune system, where it defends against viral infections, CRISPR/Cas9 has been repurposed as a revolutionary tool for precise genome manipulation across various organisms, including bacteria [4]. The CRISPR/Cas9 system works by utilizing a customizable RNA sequence called guide RNA (gRNA) to direct the Cas9 enzyme to a specific DNA sequence in the bacterial genome. Upon binding, Cas9 cleaves the DNA at the target site, enabling the deletion, modification, or replacement of genes. This precision has positioned CRISPR/Cas9 as a promising approach to addressing antibiotic resistance, specifically by targeting and eliminating resistance-conferring genes. CRISPR-based strategies can also be employed to remove resistance-carrying plasmids, destroy virulence factors, and even selectively kill resistant bacterial strains while sparing non-resistant ones. The application of CRISPR/Cas9 for combating drug-resistant bacteria is still in its early stages, but research shows great promise. Phage-delivered CRISPR systems, for example, are being developed to introduce CRISPR components into bacterial cells, specifically targeting resistance genes. In theory, this approach could reverse resistance and restore bacterial sensitivity to antibiotics [5]. Other strategies include targeting genes responsible for biofilm formation or bacterial virulence, which can help weaken the pathogens and improve the efficacy of existing antibiotic treatments. Despite these exciting developments, several challenges remain, including the efficient delivery of CRISPR/Cas9 components to bacterial populations in real-world environments, potential off-target effects, and the risk that bacteria may evolve resistance to CRISPR-based interventions. Nonetheless, CRISPR/Cas9 represents a powerful tool in the ongoing battle against antibiotic resistance and offers a new avenue for the development of next-generation antimicrobial therapies. This paper explores the molecular mechanisms that contribute to bacterial drug resistance and highlights the advances in CRISPR/Cas9-based genome editing solutions [6]. The integration of CRISPR technology into antimicrobial strategies could revolutionize the treatment of resistant infections, providing hope for overcoming one of the most pressing medical challenges of our time.

## Molecular mechanisms of drug resistance in bacteria

The molecular mechanisms of drug resistance in bacteria are diverse and complex. They can be classified into several categories, each involving different strategies that bacteria use to survive in the presence of antibiotics. Drug resistance in bacteria is a major global health issue, with mechanisms that can include: 1). Efflux pumps: These proteins actively expel antibiotics from bacterial cells, reducing drug concentration to sub-lethal levels [7]. 2). Enzymatic degradation or modification: Bacteria produce enzymes like beta-lactamases, which can deactivate antibiotics [8]. Mutations alter the bacterial components targeted by the antibiotic, rendering it less effective [9]. Biofilm formation: Bacteria in biofilms are more resistant to antibiotics due to reduced penetration and slower growth rates [10]. Genes responsible for resistance can be transferred between bacteria through plasmids, transposons, or bacteriophages [11].

**Fig. 1:**
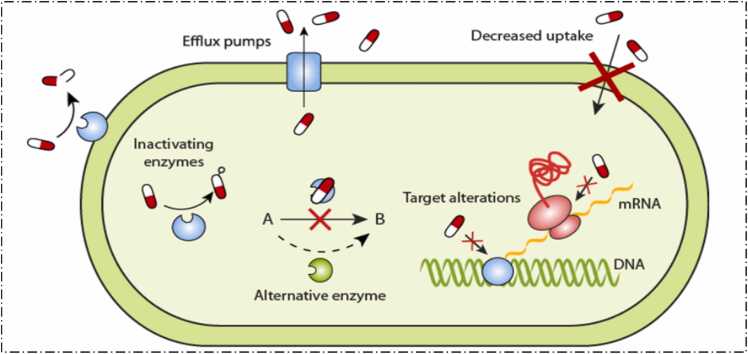
Molecular mechanism of Antibiotic-resistance in Bacteria.

The Fig. 1 represents various mechanisms through which bacteria develop resistance to antibiotics. Bacteria have specialized proteins in their membranes (shown as blue pumps) that actively expel antibiotics from the cell. This prevents the antibiotic from reaching its target concentration inside the bacterial cell, making the treatment less effective known as the Efflux pumps. The bacterial membrane acts as a barrier that limits the entry of antibiotics. Some antibiotics are unable to penetrate the bacterial cell wall or membrane due to modifications in the bacterial outer structure, leading to reduced antibiotic uptake these are commonly known for its permeability. Bacteria produce enzymes (represented by the green structure) that degrade or break down antibiotics. A common example is the production of beta-lactamases, enzymes that degrade beta-lactam antibiotics like penicillin. Bacteria can modify the molecular targets that antibiotics typically bind to, rendering the antibiotic ineffective. For example, the bacterial ribosome, where protein synthesis occurs, can undergo changes that prevent antibiotics like tetracycline from binding to it, thus halting their action. Bacteria may chemically modify antibiotics through specific enzymes, thereby inactivating the drug. In this scenario, the antibiotic's structure is altered by the bacteria, preventing it from functioning as intended. These resistance mechanisms highlight the challenges posed by antibiotic resistance, where bacteria evolve to evade the effects of antibiotics, making infections harder to treat. Understanding these processes is crucial for developing strategies to combat drug-resistant bacteria [12].

### Efflux pumps vs drug resistance bacteria

Efflux pumps are transport proteins (membrane-bound proteins) located in the bacterial cell membrane that actively expel toxic substances, including antibiotics, out of the cell. They use energy, typically in the form of ATP hydrolysis or the proton motive force, to pump out harmful substances. These pumps are not limited to antibiotics; they also expel toxic compounds, metabolic by-products, and other chemicals [13]. By reducing the intracellular concentration of the antibiotic, these pumps prevent the drug from reaching its target site within the bacterium. The AcrAB-TolC efflux pump in Escherichia coli is known to pump out a wide range of antibiotics, including tetracycline and chloramphenicol [14]. Efflux pumps can confer multi-drug resistance (MDR) as they often transport different classes of antibiotics. Efflux pumps play a significant role in the ability of bacteria to resist antibiotic treatments. They are one of the most common and versatile mechanisms of drug resistance. Efflux pumps are a major contributor to bacterial drug resistance due to their ability to: Decrease Intracellular Antibiotic Concentration: By actively removing antibiotics from the cell, efflux pumps lower the drug's concentration inside the bacterium to sub-lethal levels, preventing it from reaching its target. Many efflux pumps are capable of expelling a wide range of structurally unrelated antibiotics, leading to multi-drug resistance. This means that a single efflux pump can provide resistance against multiple classes of antibiotics [15]. Some efflux pumps have broad substrate specificity, which allows them to recognize and export different antibiotics, toxic compounds, and even dyes [16]. Bacteria with efflux pumps may develop cross-resistance, where resistance to one antibiotic leads to resistance to other drugs that are also substrates of the same pump [17].

#### Types of efflux pumps in bacteria

Bacterial efflux pumps are categorized into various types depending on their protein sequence similarity, energy source, and general structure. The major facilitator superfamily (MFS) represents one of the most extensively distributed types of efflux pumps within microbial genomes, showcasing a wide range of substrate specificities. These pumps can function as single-component transporters or be components of tripartite complexes. In Gram-negative bacteria, the resistance-nodulation-division (RND) superfamily of efflux pumps has a crucial clinical importance. For example, in Pseudomonas aeruginosa, four extensively studied multidrug efflux pump systems have been recognized: MexA-MexB-OprM, MexC-MexD-OprJ, MexE-MexF-OprN, and MexX-MexY-OprM. These pumps exhibit different substrate specificities and can be produced in excess due to various factors. Interestingly, although efflux pumps are frequently linked to antibiotic resistance, they have several physiological roles in addition to drug expulsion. These encompass bacterial adjustments to harsh conditions, the expulsion of toxins and metabolites, the creation of biofilms, and quorum sensing. This multifunctionality implies that efflux pumps have deep-rooted origins and are not exclusively developed for antibiotic resistance. In summary, bacterial efflux pumps are varied and intricate mechanisms that are essential for antibiotic resistance and numerous physiological functions. Grasping the varieties and functions of these pumps is crucial for creating methods to tackle multidrug resistance and for obtaining knowledge about bacterial behaviour in various environments. Efflux pumps can be categorized into several families based on their structure and energy source: which include, These use ATP as the energy source to transport substances. MsbA in Escherichia coli. These pumps use the proton gradient (proton motive force) to drive the export of drugs. EmrD in *Escherichia coli*. Commonly found in Gram-negative bacteria, these pumps are responsible for expelling a wide range of antibiotics. AcrAB-TolC system in *Escherichia coli*. These pumps also use the proton motive force and are involved in exporting small, hydrophobic molecules. These pumps use either sodium ions or a proton gradient for energy and are involved in expelling drugs and other toxic compounds [18].

#### Efflux pumps in gram-positive vs. gram-negative bacteria

Efflux pumps are essential in antibiotic resistance for both Gram-positive and Gram-negative bacteria, although their functions and effects vary greatly between these two bacterial groups. In Gram-negative bacteria, efflux pumps are more intricate and play a significant role in intrinsic resistance to various antibiotics, detergents, dyes, and organic solvents. These pumps are made up of three parts: an inner membrane transporter, an outer membrane channel, and a periplasmic lipoprotein, enabling them to directly discharge substrates into the external medium through both membranes. Notably, although efflux pumps have been extensively researched in Gram-negative bacteria, knowledge about solvent tolerance mechanisms in Gram-positive bacteria is still limited. Certain common mechanisms found in both Gram-positive and Gram-negative bacteria include energy-driven active efflux pumps, alterations in membrane fatty acids and phospholipids, and the creation of vesicles filled with harmful substances. Nonetheless, Gram-positive bacteria might exhibit unique physiological reactions to organic solvents that are not seen in Gram-negative bacteria. Efflux pumps in Gram-negative bacteria, especially from the resistance-nodulation-division (RND) family, play a major role in multidrug resistance and have been researched more thoroughly than those found in Gram-positive bacteria. The intricate architecture of Gram-negative bacterial cell walls, along with the existence of advanced efflux systems, renders them naturally more resilient to numerous antibiotics than Gram-positive bacteria. This distinction emphasizes the necessity for focused methods in creating efflux pump inhibitors and alternative strategies to address antibiotic resistance in both bacterial types**.** Efflux pumps in these bacteria are generally more effective because of their complex cell wall structure, which includes an outer membrane that acts as a barrier to antibiotics. The RND family pumps, in particular, form tripartite complexes that span the inner membrane, periplasmic space, and outer membrane, providing a highly efficient means to expel antibiotics directly out of the cell [19]. Although Gram-positive bacteria lack an outer membrane, they still possess potent efflux pumps (mainly from the MFS family) that help in antibiotic resistance by reducing the drug concentration within the cytoplasm [20].

#### Clinical implications

Efflux pumps significantly contribute to treatment failure in infections caused by multi-drug-resistant bacteria. Common pathogens like Escherichia coli, Pseudomonas aeruginosa, Staphylococcus aureus, and Mycobacterium tuberculosis often use efflux pumps to resist antibiotics [21]. Efflux pumps are a major obstacle in drug development because they can quickly render new antibiotics ineffective. Overcoming the action of efflux pumps is one of the key goals in designing new therapeutic agents [22].

#### Strategies to inhibit efflux pumps

Inhibiting efflux pumps has become a potential approach to address antimicrobial resistance and hinder biofilm development in bacteria. Various strategies have been investigated to block efflux pumps, encompassing the creation of small molecule inhibitors, natural substances, and synthetic materials. These efflux pump inhibitors (EPIs) may be able to reinstate the effectiveness of antibiotics by stopping the expulsion of drugs from bacterial cells. Notably, certain polymeric pharmaceutical excipients such as Tweens® and Pluronics® have shown the ability to inhibit efflux pumps, prompting research into other polymers like polysaccharides, polyethylene glycols, and dendrimers for their possible efflux pump inhibitory properties. Furthermore, new approaches such as gene silencing and inhibitory antibodies are being investigated to disrupt the expression and function of efflux pumps. In summary, the creation of efflux pump inhibitors provides a diverse strategy for addressing antimicrobial resistance. By focusing on efflux pumps, researchers seek to improve antibiotic effectiveness while also possibly decreasing bacterial virulence and hindering biofilm development. The strategic design of EPIs, enhanced by better comprehension of pump structures and regulation, along with novel strategies such as host-directed therapy and antimicrobial peptides, offers a hopeful pathway to combat efflux-mediated resistance in pathogenic bacteria.

Researchers are exploring several strategies to inhibit efflux pumps and overcome bacterial resistance: which includes the compounds designed to block the activity of efflux pumps, thereby increasing the intracellular concentration of antibiotics. Examples include reserpine and verapamil. Using antibiotics in combination with EPIs or other agents that can reduce the expression or activity of efflux pumps. Designing antibiotics that are poor substrates for efflux pumps, making it difficult for the bacteria to expel them [23]. Efflux pumps are a powerful defence mechanism that bacteria use to survive in the presence of antibiotics. Their ability to handle a broad range of substances, including multiple classes of antibiotics, makes them a significant factor in multi-drug resistance. Understanding efflux pump mechanisms and developing effective inhibitors is essential for combating antibiotic resistance and improving the efficacy of antimicrobial therapies [24].

### Enzymatic degradation or modification vs drug resistance bacteria

Enzymatic degradation or modification is another crucial mechanism by which bacteria develop resistance to antibiotics. It involves the use of bacterial enzymes that either destroy or chemically alter the antibiotic molecules, rendering them ineffective [25].

#### Enzymatic degradation: mechanism and examples

Enzymatic degradation involves the breakdown of antibiotic molecules by bacterial enzymes, preventing the drugs from reaching their target or interfering with their action. Key Mechanism: These enzymes catalyze chemical reactions that destroy the structure of the antibiotic, making it inactive [26]. Beta-lactamases: Beta-lactamases are enzymes that specifically target beta-lactam antibiotics, such as penicillins, cephalosporins, and carbapenems. They hydrolyze the beta-lactam ring, a critical structural component required for the antibiotic's activity [27]. Staphylococcus aureus and Escherichia coli produce beta-lactamase enzymes that confer resistance to a wide range of beta-lactam antibiotics. Extended- These enzymes have evolved to break down even broader classes of beta-lactam antibiotics, including third-generation cephalosporins and aztreonam, which were once thought to be resistant to degradation. Carbapenemases are specialized beta-lactamases that can hydrolyze carbapenems, which are considered last-resort antibiotics used to treat severe bacterial infections. Bacteria like *Klebsiella pneumoniae* produce carbapenemases, making infections extremely difficult to treat [28].

#### Enzymatic modification: mechanism and examples

Enzymatic modification refers to the chemical alteration of antibiotic molecules by bacterial enzymes, which reduces the antibiotic's ability to bind to its target. Key Mechanism: These modifications usually involve adding chemical groups to the antibiotic, such as acetyl, phosphate, or adenyl groups, thereby neutralizing its activity [29]. AMEs chemically modify aminoglycoside antibiotics (e.g., gentamicin, tobramycin, and kanamycin) by adding acetyl, phosphate, or adenyl groups to the drug molecule. These modifications prevent the antibiotic from binding to the bacterial ribosome, thus inhibiting its ability to interfere with protein synthesis. Bacteria like *Pseudomonas aeruginosa* and *Enterococcus* species are known to use these enzymes to inactivate aminoglycosides [30]. This enzyme acetylates chloramphenicol, a broad-spectrum antibiotic, rendering it inactive [31]. The addition of an acetyl group prevents chloramphenicol from binding to the bacterial ribosome, stopping its inhibitory effect on protein synthesis**.** *Escherichia coli* and *Salmonella* species commonly produce CAT to resist the action of chloramphenicol [32].

**Table 1:** Comparison of Enzymatic Degradation and Modification in Drug Resistance.

**Feature**	**Enzymatic Degradation**	**Enzymatic Modification**
Mechanism	Breakdown of the antibiotic structure	Chemical alteration of the antibiotic molecule[33]
Action	Destroys the active component of the drug	Reduces drug binding to its binding target[34]
Examples of enzymes	Beta-lactamases, Carbapenemases	Aminoglycoside-modifying enzymes, Chloramphenicol acetyltransferase[35]
Commonly Affected Antibiotics	Beta-lactams (penicillins, cephalosporins), Carbapenams	Aminoglycoside (gentamicin), Chloramphenicol[36]
Clinical Impact	Major role in resistance to penicillin and cephalosporin	Contributes to multi-drug resistance (MDR) bacteria[37]

**Table 2:** Comparison of Target Modification with Other Mechanisms.

**Feature**	**Target Modification**	**Other Mechanisms (Efflux Pumps, Enzymatic Degradation)**
Mechanism	Alteration of antibiotic target sites	Removal (efflux pumps) or inactivation (enzymatic degradation) of antibiotics[48]
Specificity	Usually specific to certain antibiotics	Efflux pumps may be broad-spectrum, and degrading enzymes can target specific classes[49]
Commonly Affected antibiotics	Beta-lactams, macrolides, fluoroquinolones, glycopeptides	Multiple classes including beta-lactams, aminoglycosides, Chloramphenicol[50]
Impact on Cross-Resistance	Often leads to cross-resistance within a class	Efflux pumps and enzymes can cause broader multi-drug resistance[51]

**Table 3:** Comparison of Reduced Permeability with Other Mechanisms.

**Feature**	**Reduced Permeability**	**Other Mechanisms (Efflux Pumps, Enzymatic Degradation, Target Modification)**
Mechanism	Decreased uptake of antibiotics due to altered membrane points	Removal (efflux pumps), inactivation (enzymatic degradation), or altered target sites[58]
Specificity	Mainly affects hydrophilic antibiotics	Efflux pumps may act broadly; enzymatic degradation and target modification are often drug-specific[59]
Commonly Affected Antibiotics	Beta-lactams, carbapenams, fluoroquinolones	Multiple classes, including aminoglycosides, chloramphenicol, macrolides[60]
Synergy with other Mechanisms	Often works together with efflux pumps and other mechanisms	Other mechanisms can also be combined but with different resistance strategies[61]

#### Clinical implications of enzymatic degradation or modification

The enzymatic breakdown and alteration of biomaterials hold considerable clinical relevance, especially in drug delivery, tissue engineering, and the creation of medical devices. The regulated breakdown of substances such as poly(lactic acid) (PLA) and poly(epsilon-caprolactone) (PCL) can be leveraged for specific drug delivery and scaffold decomposition in tissue engineering uses. Remarkably, the enzymatic breakdown process is affected by several elements like enzyme concentration, pH levels, temperature, and ionic strength. For example, Proteinase K effectively breaks down PLA, with an ideal temperature close to 50 °C, which exceeds the typical 37 °C found in academic sources. This information can be utilized to create materials with customized degradation characteristics for particular clinical uses. Grasping the processes of enzymatic degradation is essential for creating biodegradable materials that exhibit consistent in vivo behaviour. The capacity to regulate degradation rates via elements such as material makeup, enzyme variety, and environmental factors presents possibilities for developing sophisticated drug delivery systems and scaffolds for tissue engineering. Moreover, the enzymatic breakdown of synthetic polymers such as polyethylene presents opportunities for tackling environmental issues associated with plastic waste. Enzymatic degradation or modification leads to resistance against some of the most widely used classes of antibiotics, limiting treatment options for bacterial infections. Bacteria capable of producing these enzymes often exhibit resistance to multiple antibiotics, resulting in multi-drug-resistant strains that are more challenging to treat [38]. Genes encoding these enzymes are frequently located on plasmids, which can easily be transferred between bacteria through processes like conjugation, transformation, and transduction. This contributes to the rapid spread of resistance in bacterial populations [39].

#### Strategies to combat enzymatic degradation or modification

Methods to counteract the enzymatic breakdown or alteration of antibiotics and other therapeutic substances are essential in tackling the increasing problem of drug resistance. Various methods have been created to improve the effectiveness and stability of these substances. One approach includes utilizing inhibitors to oppose the enzymatic alteration of antibiotics. For example, certain inhibitors have been discovered that can block the enzymatic alteration of isoniazid and rifampin, affecting the treatment of multi-drug-resistant mycobacteria. In a similar manner, analogues of anhydrotetracycline have been developed and assessed as small molecule blockers of tetracycline-inactivating enzymes, demonstrating potential in restoring tetracycline efficacy against resistant bacteria. An alternative method emphasizes enhancing the metabolic stability and half-life of peptides, which are crucial in several domains such as drug discovery. Structural alterations and innovative delivery strategies have been created to improve peptides' durability against enzymatic breakdown. Cell-penetrating peptides and stapled modified peptides have shown greater stability than their parent peptides. Interestingly, certain strategies integrate various methods. For instance, inhibitors of wall teichoic acid (WTA) biosynthesis have been explored as combination therapies to enhance β-lactam effectiveness against methicillin-resistant Staphylococcus aureus (MRSA). These inhibitors focus on the WTA transporter protein TarG and have demonstrated potential efficacy as anti-MRSA β-lactam combination agents in both in vitro and animal research. Addressing enzymatic degradation or alteration of therapeutic agents necessitates a comprehensive strategy. Approaches like creating resistance mechanism inhibitors, enhancing compound stability via structural changes, and employing combination therapies present potential solutions to this issue. Ongoing research in this area is crucial to remain ahead of developing resistance mechanisms and ensure the effectiveness of both current and upcoming therapeutic agents. Compounds like clavulanic acid, sulbactam, and tazobactam are used in combination with beta-lactam antibiotics to inhibit beta-lactamase enzymes and restore the effectiveness of the drug [40]. Designing antibiotics that are not susceptible to enzymatic degradation or modification, such as beta-lactams with novel side chains that prevent beta-lactamase action. Using multiple antibiotics together to reduce the likelihood of bacteria developing resistance through enzymatic means [41]. Enzymatic degradation and modification are highly effective mechanisms that bacteria use to inactivate antibiotics. These strategies not only neutralize the drugs but also contribute significantly to multi-drug resistance (MDR), making treatment of bacterial infections more challenging. Understanding these processes is crucial for developing new approaches to inhibit bacterial enzymes and enhance the efficacy of existing antibiotics [42].

### Target modification vs drug resistance bacteria

Target modification is another critical mechanism by which bacteria develop resistance to antibiotics. This strategy involves alterations to the bacterial components that antibiotics are designed to target, making the drugs less effective or even completely ineffective.

#### Role of target modification in drug resistance

Target modification refers to the structural or chemical changes in bacterial molecules that antibiotics bind to, such as enzymes, ribosomes, or cell wall components. Mechanism: When the target site of an antibiotic undergoes modification, the antibiotic can no longer effectively interact with its target, thereby losing its ability to inhibit bacterial growth or kill the bacteria. Target modification is a major contributor to resistance against several classes of antibiotics, including beta-lactams, fluoroquinolones, macrolides, and glycopeptides. It works by: Altered target sites decrease the binding affinity of the antibiotic, making it difficult for the drug to exert its effects. Some bacteria evolve their target molecules in such a way that they maintain their normal function while becoming less susceptible to the action of the antibiotic. Changes in target sites can lead to cross-resistance, where bacteria become resistant to multiple antibiotics that act on similar targets [43].

#### Common examples of target modification in drug resistance

Beta-lactam antibiotics, like penicillins and cephalosporins, work by binding to and inhibiting PBPs, which are enzymes involved in bacterial cell wall synthesis. Some bacteria acquire mutations or new versions of PBPs with reduced affinity for beta-lactam antibiotics. Methicillin-resistant Staphylococcus aureus (MRSA) produces a modified PBP called PBP2a, which has a low affinity for beta-lactams, allowing the bacteria to continue cell wall synthesis even in the presence of these drugs [44]. This modification renders the bacteria resistant to a broad spectrum of beta-lactam antibiotics, including penicillins, cephalosporins, and even some carbapenems.

#### Ribosomal modifications in macrolide and aminoglycoside resistance

Antibiotics like macrolides (e.g., erythromycin) and aminoglycosides (e.g., gentamicin) target bacterial ribosomes to inhibit protein synthesis. Bacteria can develop mutations in ribosomal RNA (rRNA) or ribosomal proteins that prevent these antibiotics from binding effectively. Resistance to macrolides in Streptococcus pneumoniae is often due to methylation of the 23S rRNA subunit, which reduces the binding affinity of the drug to the ribosome [45]. Ribosomal modifications can lead to high-level resistance against protein synthesis inhibitors, making them ineffective against these bacterial strains.

#### DNA gyrase and topoisomerase iv modifications in fluoroquinolone resistance

Fluoroquinolones (e.g., ciprofloxacin) target DNA gyrase and topoisomerase IV, enzymes crucial for bacterial DNA replication. Mutations in the genes encoding these enzymes reduce the binding of fluoroquinolones. In Escherichia coli and Pseudomonas aeruginosa, mutations in the gyrA and parC genes alter the structure of DNA gyrase and topoisomerase IV, leading to decreased fluoroquinolone binding. Such modifications can lead to resistance against multiple fluoroquinolones, posing a challenge in treating infections caused by these bacteria [46].

#### D-Ala-D-lac substitution in vancomycin resistance

Vancomycin inhibits cell wall synthesis in Gram-positive bacteria by binding to the D-Ala-D-Ala terminus of peptidoglycan precursors. Some bacteria, like vancomycin-resistant Enterococcus (VRE), modify this target to D-Ala-D-Lac, which greatly reduces vancomycin binding. VRE uses this modification to prevent vancomycin from interfering with cell wall synthesis, making the antibiotic ineffective. This modification is a significant cause of resistance in serious hospital-associated infections where vancomycin is often used as a last-resort antibiotic [47].

#### Clinical implications of target modification in drug resistance

Target modification is a significant challenge in clinical settings because it directly affects the antibiotic's site of action, rendering some of the most commonly used drugs ineffective. Bacteria that undergo target modifications often exhibit resistance to multiple drugs in the same class, complicating treatment protocols and requiring the use of alternative or combination therapies. Target modification can arise from spontaneous mutations or acquisition of resistance genes through horizontal gene transfer, contributing to the rapid evolution of resistant strains [52]. Target modification is a powerful mechanism that bacteria use to evade the effects of antibiotics. By altering the very sites that antibiotics are designed to target, bacteria can survive and multiply even in the presence of drugs. This mechanism plays a critical role in multi-drug resistance and presents a significant challenge in treating bacterial infections. Understanding and addressing target modification is essential for developing more effective antimicrobial therapies and combating the growing threat of antibiotic resistance [53].

### Reduced permeability vs drug resistance bacteria

Reduced permeability is a mechanism by which bacteria develop resistance to antibiotics by decreasing the drug's ability to penetrate the bacterial cell. This mechanism primarily involves changes in the bacterial cell membrane or cell wall that limit the entry of antibiotics into the cell, thereby reducing their intracellular concentration [54].

#### Role of reduced permeability in drug resistance

Reduced permeability refers to the alteration of bacterial cell membranes or cell walls that impedes the influx of antibiotics into the bacterial cell. This decrease in permeability is mainly achieved by modifying or reducing the number of porin channels, which are proteins that form pores in the outer membrane of Gram-negative bacteria, allowing the passage of small molecules, including antibiotics [55]. Reduced permeability is particularly important in resistance to hydrophilic antibiotics that rely on porin channels to enter the bacterial cell, such as beta-lactams, tetracyclines, and some fluoroquinolones. The main ways it contributes to resistance include. When fewer or modified porins are present, the antibiotic cannot efficiently enter the bacterial cell, leading to sub-therapeutic concentrations of the drug within the cell. Reduced permeability often works in conjunction with other resistance mechanisms like efflux pumps or enzymatic degradation, enhancing the bacterium's overall ability to survive in the presence of antibiotics. This mechanism helps bacteria survive in hostile environments where antibiotics are present, contributing to their persistence and the spread of resistance [56].

#### Common examples of reduced permeability in drug resistance

Gram-negative bacteria have an outer membrane that acts as a barrier to antibiotics. Porins are channels in this membrane that allow the passive diffusion of molecules into the cell. By altering the expression or structure of these porins, bacteria can significantly decrease the uptake of antibiotics. *Pseudomonas aeruginosa* and *Escherichia coli* commonly reduce the expression of the OprD porin, which normally facilitates the entry of carbapenems (e.g., imipenem). This modification is a key factor in their resistance to these drugs. The reduction or loss of porins like OprD leads to a marked decrease in antibiotic susceptibility, often resulting in resistance to a broad range of beta-lactam antibiotics [57]. Mycobacteria, such as Mycobacterium tuberculosis, have a unique, thick, and waxy cell wall rich in mycolic acids that acts as a strong permeability barrier. This cell wall structure limits the entry of many antibiotics, contributing to their intrinsic resistance. The reduced permeability of the cell wall in mycobacteria is one of the reasons why tuberculosis treatment requires long durations and combinations of multiple antibiotics.

#### Reduced permeability in gram-positive vs. gram-negative bacteria

Reduced permeability is more prominent in Gram-negative bacteria due to their outer membrane, which acts as an additional barrier to antibiotic entry. The outer membrane is equipped with porins, and any changes in these porins can drastically reduce antibiotic uptake. Although Gram-positive bacteria do not have an outer membrane, they can still develop resistance through modifications in their thick peptidoglycan layer or by producing molecules that interact with the cell membrane to hinder drug entry.

#### Clinical implications of reduced permeability in drug resistance

Reduced permeability plays a significant role in multi-drug-resistant bacteria, particularly when combined with other mechanisms like efflux pumps and enzymatic degradation. Bacteria that employ reduced permeability are often resistant to several classes of antibiotics, making them difficult to treat and control, especially in healthcare-associated infections. Reduced drug uptake can lead to persistent infections that are harder to eradicate, increasing the risk of relapse after treatment.

Reduced permeability is a crucial mechanism that bacteria use to evade the effects of antibiotics by limiting the drug's entry into the cell. This mechanism is especially important in Gram-negative bacteria, which have an outer membrane that can be modified to reduce antibiotic penetration. When combined with other resistance strategies, reduced permeability significantly contributes to multi-drug resistance and the challenge of treating bacterial infections. Understanding this mechanism is essential for developing new antibiotics and strategies to enhance drug uptake in resistant bacterial strains.

### Biofilm formation & drug resistance bacteria: the connection

Reduced Antibiotic Penetration: Biofilms form a protective barrier that prevents antibiotics from reaching all bacterial cells within the biofilm. This physical obstruction makes it difficult for drugs to fully eradicate the bacteria. Slow Growth Rates: Bacteria within biofilms often enter a slow-growing or dormant state. Many antibiotics target actively dividing cells, so this slow growth can make the bacteria less susceptible to treatment. Quorum Sensing: Biofilm bacteria communicate using quorum sensing, which can regulate the expression of resistance genes [62]. This can lead to coordinated responses to stress, including antibiotic pressure. Genetic Exchange: The close proximity of bacteria within biofilms facilitates the transfer of genetic material, including antibiotic resistance genes, through horizontal gene transfer. This means resistant bacteria within the biofilm can share resistance traits with neighbouring cells. Persistent Cells: Biofilms often harbour “persister” cells, which are a small subpopulation of bacteria that can survive antibiotic treatment due to their dormant state. After antibiotic treatment is over, these cells can repopulate the biofilm, leading to chronic or recurrent infections [63].

#### Clinical relevance

Chronic Infections: Biofilms are implicated in many chronic infections, such as those associated with cystic fibrosis, urinary tract infections, endocarditis, and infections involving medical devices (e.g., catheters, prosthetic joints) [64]. These infections are notoriously difficult to treat due to the combination of biofilm formation and drug resistance. Increased Antibiotic Usage: The presence of biofilms often requires higher doses or longer courses of antibiotics, which can further drive the selection for resistant bacteria.[65] Key Takeaways: Biofilm formation and drug resistance are both strategies that increase bacterial survival under hostile conditions, especially during antibiotic treatment. Biofilm-embedded bacteria are more resistant to antibiotics than their planktonic counterparts, primarily due to the protective nature of the biofilm matrix, reduced growth rates, and genetic exchange [66]. Addressing infections involving biofilms often requires a combination of strategies, including high-dose or combination antibiotic therapy, biofilm-disrupting agents, and in some cases, mechanical removal (e.g., surgical debridement) [67].

### Horizontal gene transfer (HGT) vs. drug resistance in bacteria

The spread of antibiotic resistance in bacteria, particularly in healthcare settings, is largely dependent on horizontal gene transfer (HGT). Multidrug-resistant (MDR) pathogens may arise as a result of HGT's ability to quickly acquire new DNA sequences, such as antimicrobial resistance genes (ARGs). This problem is especially concerning when it comes to hospital-acquired infections because resistant *Acinetobacter baumannii* can cause death rates to rise to 19–54 %. It's interesting to note that while HGT promotes the spread of resistance, some bacterial defense mechanisms might be able to slow this process down. For instance, bacteria's CRISPR loci may act as a modifiable barrier to HGT, preventing the in vivo development of virulence. However, bacterial pathogens may lose CRISPR due to strong selection for virulence or antibiotic resistance. Additionally, it has been shown that *Acinetobacter baylyi*-induced bacterial predation greatly increases cross-species HGT by several magnitudes, further complicating the dynamics of resistance transmission. HGT works is crucial to combating the spread of bacterial drug resistance. Numerous mechanisms for HGT, including conjugation, transformation, transduction, and membrane vesicles, have been discovered recently. Horizontal gene transfer (HGT) and drug resistance are closely linked phenomena in bacterial evolution. While HGT is a process by which bacteria acquire genetic material from other organisms (rather than inheriting it from parent cells), drug resistance in bacteria refers to their ability to survive exposure to antibiotics [68]. These two processes are often interconnected, as HGT facilitates the spread of antibiotic resistance genes among bacterial populations. Horizontal gene transfer is the movement of genetic material between organisms other than through direct inheritance from parent to offspring. In bacteria, this is one of the primary mechanisms for acquiring new traits, including antibiotic resistance [69].

#### Mechanisms of horizontal gene transfer (HGT)

Bacteria take up free DNA from their environment (often from lysed cells) and integrate it into their own genome. This process can allow bacteria to acquire resistance genes from dead or dying bacteria [70]. Bacteriophages (viruses that infect bacteria) transfer DNA between bacterial cells. During viral infection, bacterial DNA, including antibiotic resistance genes, can be packaged into phage particles and transferred to other bacteria. Direct transfer of DNA between bacteria through a physical connection, usually via a pilus (a bridge-like structure). Plasmids (small, circular DNA molecules) are often transferred in this manner. Many plasmids carry multiple antibiotic resistance genes, which can spread rapidly within and across bacterial species [71].

#### Significance of HGT

HGT allows bacteria to acquire new traits without waiting for mutations to occur in their own genomes [72]. Bacteria can quickly adapt to new environments, including the presence of antibiotics, by acquiring resistance genes from other species or strains. HGT plays a key role in the spread of multidrug resistance (MDR), where bacteria carry resistance to several antibiotics [73].

#### HGT and drug resistance: the connection

Spread of Resistance Genes: HGT is the primary driver of the spread of antibiotic resistance in bacterial populations. Resistance genes can spread between bacteria of the same species or even between different species [74]. This is especially problematic in environments like hospitals, where different bacterial species coexist and are exposed to antibiotics. Resistance Plasmids: R-Plasmids (Resistance Plasmids): Many antibiotic resistance genes are carried on plasmids, which are easily transferred between bacteria via conjugation [75]. These plasmids often carry genes for resistance to multiple antibiotics, making bacteria highly resistant to a broad range of treatments. Antibiotic Pressure: The use of antibiotics creates selective pressure, favouring bacteria that have acquired resistance genes. Under these conditions, bacteria that receive resistance genes through HGT have a survival advantage, allowing them to proliferate and spread these traits [76].

Multidrug-Resistant Bacteria: Superbugs: Many multidrug-resistant bacteria, such as Methicillin-resistant Staphylococcus aureus (MRSA) or Extended-Spectrum Beta-Lactamase (ESBL)-producing bacteria, owe their resistance to the acquisition of multiple resistance genes via HGT. These organisms are challenging to treat due to their resistance to multiple antibiotic classes [77]. Gene Cassettes and Integrons: Integrons are genetic elements that can capture and carry multiple resistance genes in the form of gene cassettes [78]. These cassettes can be transferred between bacteria through HGT, further facilitating the spread of resistance.

#### Clinical relevance

Hospitals and healthcare settings are hotspots for the spread of drug-resistant bacteria due to the frequent use of antibiotics and the close proximity of different bacterial species. HGT accelerates the emergence and spread of multidrug-resistant infections in these environments [79]. The rapid dissemination of antibiotic resistance through HGT has led to the global spread of resistant pathogens, such as carbapenem-resistant Enterobacteriaceae (CRE) and vancomycin-resistant Enterococci (VRE), which are difficult to treat with existing antibiotics [80].

## CRISPR/Cas9-based genome editing solutions for drug-resistant bacteria

**Fig. 2:**
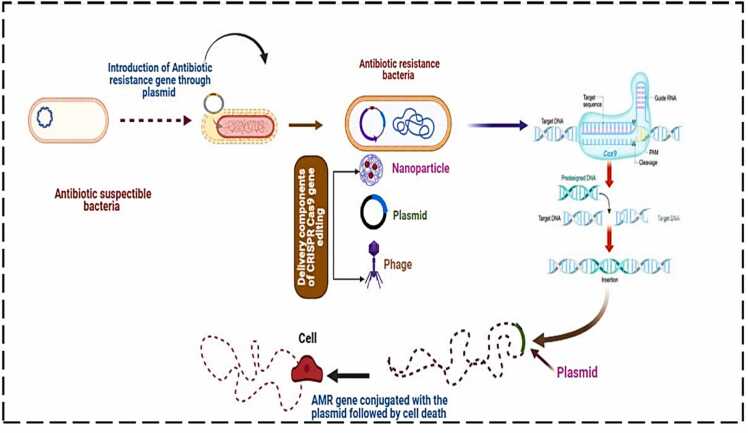
CRISPR-Cas9 Mediated Resensitisation and Targeted Cell Death in Antibiotic-Resistant Bacteria.

The CRISPR/Cas9 system, originally discovered as part of the bacterial immune system, has become a powerful tool for precise genome editing. Recently, it has been explored to combat antibiotic resistance in bacteria [81]. CRISPR (Clustered Regularly Interspaced Short Palindromic Repeats) are DNA sequences in bacteria and archaea that defend against foreign DNA, while Cas9 is an enzyme that cuts DNA at specific locations, guided by a customizable RNA sequence (gRNA) targeting specific bacterial genome sequences [82]. CRISPR/Cas9 can target and modify genes, providing a solution for antibiotic resistance. By cutting resistance genes in drug-resistant bacteria, CRISPR could make the bacteria sensitive to antibiotics again [83]. For example, targeting β-lactamase enzymes, which confer resistance to penicillin, could restore bacterial sensitivity. Many resistance genes are carried on plasmids small DNA circles separate from the bacterial chromosome [84]. CRISPR/Cas9 can be programmed to destroy these plasmids, preventing the spread of resistance genes between bacteria, such as those carrying carbapenem or ESBL resistance traits shown in Fig. 2 [85].

CRISPR/Cas9 can also be applied in a "gene drive" system, spreading throughout a bacterial population to selectively kill drug-resistant bacteria while sparing susceptible ones. It can disable virulence genes, such as those responsible for biofilm formation, reducing infection and enhancing antibiotic efficacy [86]. Another approach involves phage-delivered CRISPR, where bacteriophages (viruses that infect bacteria) introduce CRISPR components to remove resistance genes in specific bacteria like Escherichia coli and Pseudomonas aeruginosa [87]. CRISPR/Cas9 offers precision, targeting resistance genes without harming other bacterial genome parts, and can restore bacterial sensitivity to antibiotics by removing resistance genes [88]. It reduces selective pressure compared to broad-spectrum antibiotics, potentially slowing the emergence of new resistance mechanisms [89]. However, delivering CRISPR/Cas9 to bacterial cells in real-world environments poses a challenge. Phage-based delivery systems and nanoparticles are being explored but require further development [90]. Other concerns include off-target effects, resistance to CRISPR (where bacteria evolve to avoid being targeted), and immune responses against CRISPR or its delivery systems. Regulatory and ethical concerns regarding the long-term ecological impact of altering bacterial populations also need addressing. Several preclinical studies have shown success in targeting and removing resistance genes in vitro. Phage-delivered CRISPR systems have eliminated resistance genes in bacteria like Escherichia coli and Staphylococcus aureus in lab settings. Startups are exploring the commercialization of CRISPR-based antimicrobials targeting resistant bacteria [91]. CRISPR/Cas9 holds great promise for combating antibiotic resistance by targeting resistance genes, removing plasmids, and reducing bacterial virulence. However, challenges such as delivery, off-target effects, and resistance must be addressed before widespread clinical use. Combining CRISPR-based genome editing with other antimicrobial strategies could significantly address drug-resistant bacterial infections.

### CRISPR: approaches to combat multidrug-resistant bacteria

One of the most potent tools in biotechnology today is the CRISPR (Clustered Regularly Interspaced Short Palindromic Repeats) system, which was first identified as an adaptive immunological mechanism in bacteria. Its capacity to accurately alter genetic material has created ground-breaking opportunities for the fight against microorganisms that are resistant to multiple drugs (MDR) [92]. CRISPR is a promising tool to combat antimicrobial resistance (AMR) because researchers are using its gene-editing capabilities to target and eradicate antibiotic resistance genes in novel ways.

### CRISPR-cas systems: a new paradigm for antimicrobial therapy

**Fig. 3:**
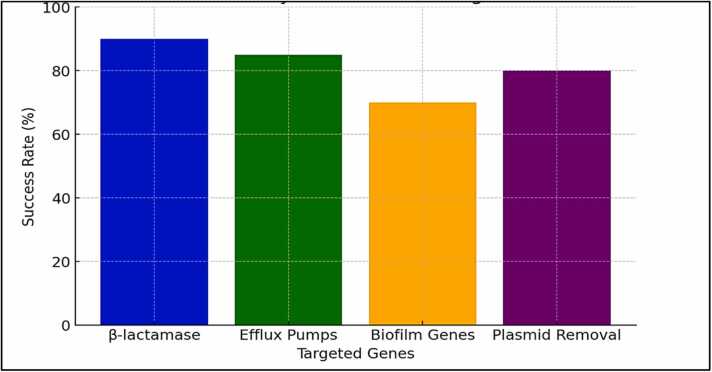
Success rates of CRISPR systems in reversing antibiotic resistance.

In order to target and cut particular DNA regions, CRISPR systems depend on the Cas (CRISPR-associated) proteins, like Cas9. Researchers can employ Cas proteins to target and cleave antibiotic resistance genes in bacteria by creating CRISPR sequences that match bacterial DNA. The success rate of CRISPR systems in reversing antibiotic resistance is illustrated in Fig. 3. This will make the infections susceptible to drugs once more. In contrast to conventional antibiotics, the most widely utilized method, CRISPR-Cas9, offers a novel mode of action by precisely targeting genomic regions that encode resistance mechanisms [93].

**Fig. 4:**
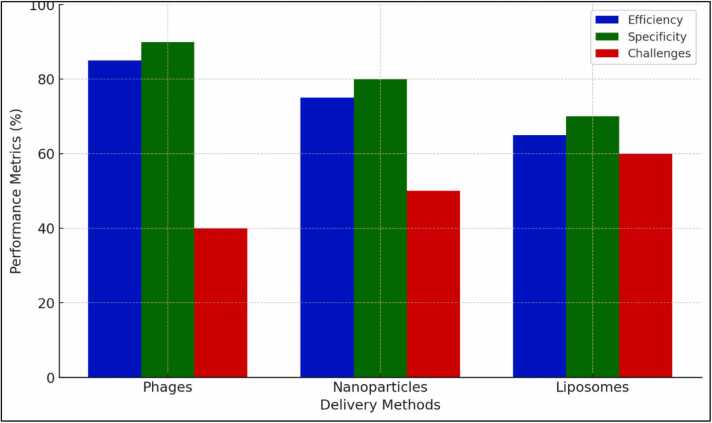
Comparison of CRISPR delivery methods.

The Fig. 4 shows the comparison of CRISPR delivery methods.

#### Targeting antibiotic resistance genes

Genes that encode β-lactamases, efflux pumps, or modified target proteins are examples of antibiotic resistance genes that can be specifically targeted by CRISPR engineering. CRISPR re-sensitizes the bacteria to antibiotics by deleting these resistance genes and interfering with their function. Disruption of β-lactamase Genes: β-lactamase genes, which provide resistance to β-lactam antibiotics, have been targeted by CRISPR-Cas systems. The CRISPR system can stop the synthesis of β-lactamase enzymes by targeting cleaving these genes, which will make β-lactam medications like cephalosporins and penicillins more effective [94]. The comparison of CRISPR efficacy against resistance mechanisms are tabulated in Table 4. Targeting Efflux Pump Genes: To lower intracellular drug concentrations, many bacteria employ efflux pumps to remove antibiotics from the cell. The genes encoding these efflux pumps can be deleted using CRISPR, increasing the susceptibility of bacteria to certain antibiotics [95].

**Table 4:** Comparison of CRISPR Efficacy against resistance mechanisms.

Resistance Mechanism	CRISPR Target species	Effectiveness (in vitro)	Role of the CRISPR gene
Β-Lactamase Genes	*TEM−1 in *E.coli*	> 90 %	Restores Susceptibility to ampicillin
Efflux Pump Genes	^$^AcrAB-TolC in *E.coli*	80 −95 %	Increases the intracellular drug levels
Target Site Modifications	DNA gyrase in *E.coli*	85 %	Resinststes fluoroquinolone activity

$ AcrAB-TolC, one of the efflux pumps, constitutively expressed in Escherichia coli, is composed of the outer membrane protein TolC, the inner membrane transporter AcrB, and the periplasmic adaptor protein

* TEM-1- a class A enzyme and is the most common plasmid-encoded β-lactamase in Gram- negative bacteria

**Fig. 5:**
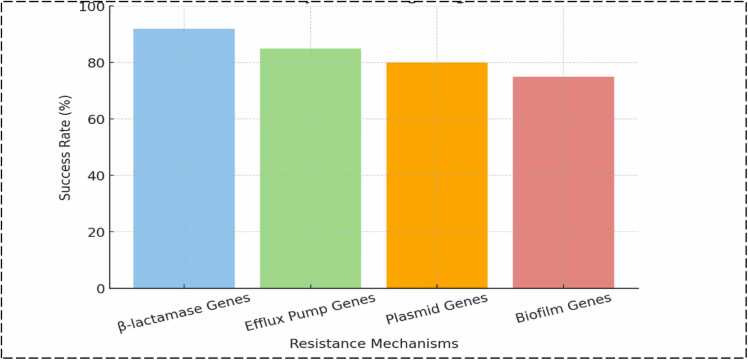
Success rates of CRISPR-Cas Systems targeting antibiotic resistance genes.

#### CRISPR-based bacteriophage therapy

To specifically target and eliminate MDR bacteria, a revolutionary strategy combines CRISPR systems with bacteriophages, which are viruses that infect bacteria. Through the direct delivery of CRISPR machinery into bacterial cells by bacteriophages, resistance genes and other vital genes necessary for bacterial life can be snipped. MDR bacteria with phages modified to deliver CRISPR components, CRISPR-Cas systems that specifically target resistance genes are introduced. Targeting drug-resistant *Klebsiella pneumoniae* and *Escherichia coli* has proven to be effective in experimental settings. This method can eradicate resistant bacteria while sparing commensal (non-pathogenic) bacteria by delivering CRISPR machinery straight to the infection site. CRISPR-based phages, which can be engineered to both kill and duplicate bacteria, the treatment can spread throughout bacterial populations. This self-amplifying nature makes CRISPR-phages an effective tool in treating localized infections caused by resistant bacteria [96].

#### Gene drives for controlling resistant bacterial populations

Gene drives, a process in which certain genetic components are engineered to spread themselves throughout bacterial populations, could be produced by CRISPR. Antibiotic resistance features can be reversed and bacteria made more antibiotic-susceptible by using gene drives to disseminate susceptibility genes. Spreading antibiotic specificity means introducing a susceptibility gene through a CRISPR-based gene drive, resistance genes could be prevented from spreading throughout a bacterial community. This strategy might be especially helpful in settings where resistance genes are common or for infections acquired in hospitals. Population Suppression generally is defined as interfering with genes necessary for bacterial growth or pathogenicity, CRISPR can be used to create gene drives that suppress bacterial populations in addition to those that propagate susceptibility [97]. The Table 5. shows the advantages and limitations of CRSPR – based gene drives.

**Table 5:** Advantages and limitations of CRSPR – baserd gene drives.

**S. No**	**Advantage**	**Limitation**
1	Reverse resistance in populations	Ecological impact of gene spread
2	Potential to suppress pathogens	Risk of unintended off target effects
3	Efficient dissemination of traits	Regulatory and ethical considerations

#### CRISPRi: transcriptional silencing of resistance genes

In a variety of organisms, CRISPR interference (CRISPRi) has become a potent tool for transcriptionally silencing resistance genes. The MAB_0055c gene in *Mycobacterium* abscesses was silenced using CRISPRi, increasing the bacteria's vulnerability to the antibiotic rifamycin [98]. This illustrates how CRISPRi can be used to research drug resistance mechanisms and find novel targets for treatment. Additionally, CRISPRi has been modified for use in vivo. Mice's Pcsk9 gene was silenced using a Staphylococcus aureus Cas9-based repressor (dSaCas9KRAB), which led to a sustained decrease in serum Pcsk9 and cholesterol levels [99]. By eliminating the cas3 gene, the endogenous Type I-E CRISPR-Cas system in *Escherichia coli* was modified for programmable transcriptional repression [100]. Many endogenous Type I systems can be easily transformed into transcriptional regulators using this method. It's interesting to note that CRISPRi has been used to silence genes linked to antibiotic resistance in environmental contexts. Cas9/sgRNAs were delivered via nitrogen-doped carbon (NCDs) to target several "high-risk" antibiotic resistance genes in E. coli, attaining long-term target gene removal and soil resensitisation of antibiotic-resistant bacteria [101]. This demonstrates how CRISPRi may be used to reduce antibiotic resistance in farming systems. To sum up, CRISPRi has shown itself to be a useful and efficient tool. Antibiotic resistance can be studied and addressed in clinical and environmental contexts with its potential to target multiple genes at once and achieve long-term silencing. A catalytically inactive Cas protein (dCas9) is used in CRISPR interference (CRISPRi), a variant of CRISPR technology, to attach to DNA without cleaving it. CRISPRi can silence antibiotic resistance genes without changing the genome by preventing the transcription of target genes. For reversible inhibition of resistance characteristics, this makes CRISPRi a desirable alternative. Silencing β-lactamase Genes: Genes that encode β-lactamases and other antibiotic-resistant genes can be silenced using CRISPRi. CRISPRi decreases the synthesis of enzymes that break down antibiotics by blocking the transcription of these genes, leaving the bacteria susceptible to therapy. The role of targeting efflux pump and regulatory genes shows that CRISPRi can be used to silence regulatory genes that govern the expression of resistance mechanisms, in addition to direct resistance genes. Transcriptional regulators are the target CRISPRi can downregulate multiple resistance pathways simultaneously [102]. The comparison of the various CRISPR Cas 9 and Cas13 are tabulated in Table 6.

**Table 6:** Comparison of CRISPRi, Cas9 and Cas13.

**Feature**	**CRISPRi (dCas9)**	**CRISPR (Cas9)**	**CRISPR (Cas13)**
Target molecule	DNA (Transcriptional block)	DNA (Cleavage)	RNA (Cleavage)
Reversibility	Reversible	Permanent	Reversible
Risk of Off target effects	Low	Medium	Low

#### CRISPR-Cas13: targeting bacterial RNA

The CRISPR-Cas13 system targets RNA, providing an alternative strategy to fight MDR bacteria, whereas CRISPR-Cas9 and related systems mostly target DNA. By cleaving bacterial RNA, Cas13 can post-transcriptionally interfere with the expression of resistance genes. Without changing the bacterial DNA, this can be a very efficient way to eliminate resistance characteristics [103]. Post-Transcriptional Gene Silencing: Antibiotic resistance genes' mRNA transcripts can be targeted by CRISPR-Cas13 to stop them from being translated into useful proteins. This method offers a versatile means of temporarily blocking resistance processes, enabling the use of antibiotics in combination treatments. Two Systems of CRISPR-Cas9/Cas13: Some methods guarantee thorough destruction of resistance genes at the DNA This dual approach could improve the effectiveness of therapies targeting resistant pathogens [104].

#### Challenges and considerations

Although CRISPR has revolutionary potential, a number of issues need to be resolved before it can be widely used as a treatment for MDR bacteria is the delivery systems which is found to be one of the biggest challenges is getting CRISPR components to the infection site efficiently. Although bacteriophages, liposomes, and nanoparticles are now used to distribute CRISPR systems, research is still crucial to optimize delivery for systemic infections. The next prime challenge is the mechanisms of bacterial evasion in which bacteria may develop defenses against CRISPR-based therapies, such as anti-CRISPR protein acquisition or mutations in the CRISPR target regions. By employing phages that evolve alongside bacteria or by targeting many resistance genes at once, researchers are looking into ways to reduce these dangers. The major considerations lie in the regulatory and ethical Issues where the application of gene-editing technologies, especially gene drives, presents moral questions about potential ecological consequences, off-target effects, and horizontal gene transfer. Careful consideration of these factors is required before CRISPR can be deployed widely.

### Future prospects of CRISPR

With its revolutionary impact on genome editing, CRISPR technology has opened up previously unheard-of avenues for research and therapeutic applications in a wide range of fields. With continuous advancements aimed at resolving present constraints and broadening its possible applications, CRISPR has a bright future. CRISPR has enormous potential for crop improvement in agriculture, especially for creating transgenic-free edited plants that don't contain foreign DNA. Numerous nations have already granted regulatory approval for this strategy, opening the door for a broader adoption of CRISPR-based crop breeding innovations. Through increased yield, improved resistance to biotic and abiotic stresses, and plant adaptation to changing climate conditions, the technology is anticipated to be instrumental in tackling the challenges of global food security. Although CRISPR has been extensively used in many different organisms, it has not been widely used in some crops, such as wheat, because of issues like the complexity of the hexaploidy genome and tissue culture resistance. On the other hand, new developments—like the publication of high-quality reference genomes—should hasten the use of CRISPR in these crops. In virology, CRISPR/Cas9 holds promise for identifying gene function, comprehending the pathophysiology of viruses, and creating vaccines, especially against large DNA viruses. The worldwide health epidemic caused by MDR bacteria can be addressed in a revolutionary way with CRISPR technologies. CRISPR-based treatments may prove to be crucial weapons in the fight against AMR if delivery methods, safety, and specificity continue to improve. CRISPR libraries that target every known gene linked to antibiotic resistance, a universal tool for battling resistance in a variety of bacterial species may be developed. Combining CRISPR editing systems with bacteriophage therapy may result in highly specific, self-replicating therapies that can overcome resistance without harming the microbiome. Precision medicine could be achieved in the future by using CRISPR to create customized antimicrobial treatments based on the particular resistance genes found in a patient's bacterial infection. Through gene editing, transcriptional silencing, and phage-mediated delivery systems, CRISPR technology presents an unparalleled opportunity to tackle bacteria that are resistant to drugs. CRISPR is a new and extremely specific method for restoring antibiotic efficacy and offering substitutes for traditional antimicrobials by precisely identifying and inhibiting antibiotic resistance processes. CRISPR is positioned to be a key component of antimicrobial therapy in the future because to continuous research into broad-spectrum applications, safety precautions, and delivery methods.

## Conclusion

Antibiotic resistance in bacteria represents an urgent global health crisis, driven by complex molecular mechanisms such as genetic mutations, efflux pumps, enzymatic degradation, target site modifications, biofilm formation, and horizontal gene transfer (HGT). These mechanisms enable bacteria to rapidly evolve and acquire multidrug resistance, rendering conventional antibiotics ineffective. As the prevalence of drug-resistant bacteria continues to rise, traditional therapeutic approaches are becoming increasingly inadequate. The advent of CRISPR/Cas9-based genome editing offers a novel and highly promising solution to combat drug resistance in bacteria. CRISPR/Cas9 provides unprecedented precision in targeting and modifying specific genes, including those responsible for antibiotic resistance. Through targeted deletion, disruption, or modification of resistance genes, this technology has the potential to reverse resistance and restore bacterial sensitivity to antibiotics. Additionally, CRISPR/Cas9 can eliminate plasmids that carry resistance genes, target bacterial virulence factors, and selectively kill resistant bacteria while sparing non-resistant strains. The combination of CRISPR/Cas9 with phage-based delivery systems further enhances its potential as a targeted antimicrobial therapy.

Despite the challenges of delivering CRISPR/Cas9 in clinical settings, mitigating off-target effects, and the possibility of bacterial resistance to CRISPR itself, ongoing research is advancing these strategies closer to real-world applications. CRISPR-based antimicrobials represent a powerful addition to the arsenal against drug-resistant pathogens, offering a targeted and precise approach to overcome resistance and improve patient outcomes. In conclusion, the integration of CRISPR/Cas9 technology into antimicrobial strategies holds immense promise for addressing the global challenge of antibiotic resistance. With continued advancements, CRISPR/Cas9 could revolutionize the treatment of drug-resistant bacterial infections, offering a new hope for curbing the rise of superbugs and restoring the efficacy of antibiotics in modern medicine.

## Ethics statement

Not applicable.

## Funding

The corresponding author thank the PSG Management for providing infrastructure and covering the expenses for the other consumables.

## CRediT authorship contribution statement

KEV- Writing Original draft preparation, conceptualization, Investigation, Data analysis, PVK- Data conceptualization, Supervision, Resources. JC-, Writing and Data conceptualization. RJ- Data conceptualization, editing.

## Declaration of Competing Interest

The authors declare the following financial interests/personal relationships which may be considered as potential competing interests: K.E.Vivekanandan reports financial support was provided by PSG College of Arts and Science. If there are other authors, they declare that they have no known competing financial interests or personal relationships that could have appeared to influence the work reported in this paper.

## Data Availability

All datasets generated or analysed during this study are included in the manuscript.

## References

[1] Ventola C.L. (2020). The antibiotic resistance crisis: part 1: causes and threats. Pharm. Ther..

[2] Murray C.J., Ikuta K.S., Sharara F., Swetschinski L., Aguilar G.R., Gray A. (2022). Global burden of bacterial antimicrobial resistance in 2019: a systematic analysis. Lancet.

[3] Tacconelli E., Carrara E., Savoldi A., Harbarth S., Mendelson M., Monnet D.L. (2022). Discovery, research, and development of new antibiotics: the WHO priority list of antibiotic-resistant bacteria and tuberculosis. Lancet Infect. Dis..

[4] Wright G.D. (2022). Mechanisms of resistance to antibiotics. Curr. Opin. Chem. Biol..

[5] Dy R.L., Richter C., Salmond G.P.C., Fineran P.C. (2020). Remarkable mechanisms in microbes to resist phage infections. Annu Rev. Virol..

[6] Álvarez B., Fernández L., Gutiérrez D., Iglesias B., Rodríguez A., García P. (2021). Phage therapy as a promising approach to reduce antibiotic use in animal production. Antibiotics.

[7] Goudarzi H., Eslami M., Farahani I., Hasanpour S., Mirzazadeh A., Shahrokh S. (2022). CRISPR-Cas system in the antibiotic-resistant bacteria: a novel approach to combat multidrug-resistance. Front Microbiol.

[8] Blair J.M.A., Webber M.A., Baylay A.J., Ogbolu D.O., Piddock L.J. (2022). Molecular mechanisms of antibiotic resistance. Nat. Rev. Microbiol.

[9] Li X.Z., Plésiat P., Nikaido H. (2021). The challenge of efflux-mediated antibiotic resistance in Gram-negative bacteria. Clin. Microbiol Rev..

[10] Hoiby N., Ciofu O., Bjarnsholt T. (2020). Pseudomonas aeruginosa biofilms in cystic fibrosis. Future Microbiol.

[11] von Wintersdorff C.J.H., Penders J., van Niekerk J.M., Mills N.D., Majumder S., van Alphen L.B. (2020). Dissemination of antimicrobial resistance in microbial communities through horizontal gene transfer. Front Microbiol..

[12] Du D., Wang-Kan X., Neuberger A., van Veen H.W., Pos K.M., Piddock L.J.V. (2021). Multidrug efflux pumps: structure, function and regulation. Nat. Rev. Microbiol.

[13] Schuster S., Vavra M., Kern W.V. (2020). Evidence of a role of the multidrug efflux pump AcrAB-TolC in resistance to chlorhexidine in Escherichia coli. J. Antimicrob. Chemother..

[14] Li X.Z., Plesiat P., Nikaido H. (2022). Efflux-mediated drug resistance in bacteria: an update. Drugs.

[15] Hassan K.A., Islam B., Liu Q., Li L., Chan C.L., Jackson S.M. (2020). Broad substrate specificity of the MATE multidrug efflux pump NorM from Pseudomonas aeruginosa. Antimicrob. Agents Chemother..

[16] Thakur V., Uniyal A., Tiwari V. (2021). Efflux pump inhibitors for bacterial infections: what is next?. Future Microbiol.

[17] Nikaido H. (2021). Structure and mechanism of RND-type multidrug efflux pumps. Adv. Enzym. Relat. Areas Mol. Biol..

[18] Gupta A., Dai X., Chalmers J.J., Shah M. (2020). Mechanisms of resistance against the efflux pump AcrAB-TolC in Escherichia coli. ACS Infect. Dis..

[19] Hegstad K., Nilsen R.M., Marvig R.L., Espedal P., Foss S., Simonsen G.S. (2020). Increased expression of efflux pumps in Pseudomonas aeruginosa biofilms. Micro Pathog..

[20] Lomovskaya O., Zgurskaya H.I., Totrov M., Watkins W.J. (2022). Efflux pumps as targets for new antibacterial therapies. Biochem Pharm..

[21] Peleg A.Y., Hooper D.C. (2020). Hospital-acquired infections due to gram-negative bacteria. N. Engl. J. Med.

[22] Schindler B.D., Kaatz G.W. (2021). Multidrug efflux pumps in Firmicutes: an evolving resistance mechanism. Future Microbiol.

[23] Reygaert W.C. (2021). Efflux pump inhibitors: targeting the bacterial response to antibiotic exposure. J. Antimicrob. Chemother..

[24] Nikaido H., Pagès J.M. (2022). Broad-specificity efflux pumps and their role in multidrug resistance of Gram-negative bacteria. FEMS Microbiol Rev..

[25] Lomovskaya O., Watkins W.J. (2021). Efflux pumps: their role in antibacterial drug discovery. Curr. Med Chem..

[26] Chaudhary A.S. (2020). A review of global initiatives to fight antibiotic resistance and recent antibiotics' discovery. Acta Pharm. Sin. B.

[27] Ramirez M.S., Tolmasky M.E. (2020). Aminoglycoside modifying enzymes. Drug Resist Updat.

[28] Vakulenko S.B., Mobashery S. (2021). Versatility of aminoglycosides and prospects for their future. Clin. Microbiol Rev..

[29] Davis J., Tomaras A.P., Roemer T. (2022). Antimicrobial resistance: the roles of gene silencing, enzyme regulation, and plasmid biology. Curr. Opin. Microbiol.

[30] Schwarz S., Fiedler S., Johnson A.P., Woodford N. (2020). Acquired resistance to chloramphenicol, quinolones, and streptogramins. Vet. Microbiol.

[31] Davis J., Tomaras A.P., Roemer T. (2022). Antimicrobial resistance: the roles of gene silencing, enzyme regulation, and plasmid biology. Curr. Opin. Microbiol.

[32] Garneau-Tsodikova S., Labby K.J. (2020). Mechanisms of resistance to aminoglycoside antibiotics: overview and perspectives. MedChemComm.

[33] Papp-Wallace K.M., Endimiani A., Taracila M.A., Bonomo R.A. (2020). Carbapenems: Past, present, and future. Antimicrob. Agents Chemother..

[34] Livermore D.M. (2020). Beta-lactamase-mediated resistance in gram-negative bacteria. Clin. Microbiol Infect..

[35] Kato H., Hayashi M., Shibata N. (2021). Identification of aminoglycoside-modifying enzymes in clinical isolates of Enterobacteriaceae: focus on the importance of the 3-N-acetyltransferase gene. Micro Drug Resist.

[36] Zhang Y., Yao Y., Zhang R. (2021). Mechanisms of chloramphenicol resistance and the presence of chloramphenicol acetyltransferase in Salmonella spp. in China. Antibiot. (Basel).

[37] Uddin N., Zubair M., Siddiqui K., Bafakeeh O., Alharbi T. (2022). Enzymatic resistance mechanisms in gram-negative bacteria: an overview of the role of beta-lactamases, aminoglycoside-modifying enzymes, and fluoroquinolone-resistance genes. Int J. Antimicrob. Agents.

[38] Rodriguez-Bano J., Moya B., Alcala L. (2021). Risk factors for acquisition of extended-spectrum β-lactamase-producing Escherichia coli in hospitalized patients. Clin. Microbiol Infect..

[39] Woodford N., Wareham D.W. (2022). Epidemiology of resistance among Enterobacteriaceae: implications for therapy. Infect. Dis. Clin. North Am..

[40] Livermore D.M., Woodford N. (2022). The beta-lactamase threat in the 21st century. J. Intern Med.

[41] Wang Y., Yang C., Zhang S. (2021). Strategies for the development of beta-lactam antibiotics that are resistant to beta-lactamases. J. Antibiot..

[42] Lee M., Peleg A.Y. (2021). Combination therapy for the treatment of multidrug-resistant infections. Infect. Dis. Clin. North Am..

[43] Kato H., Yamada K., Tada T. (2020). Penicillin-binding protein 2a is an important factor for the survival of methicillin-resistant Staphylococcus aureus in the presence of β-lactam antibiotics. J. Infect. Chemother..

[44] Chisholm S.A., Beal J., Wyllie S. (2020). Methylation of 23S rRNA in macrolide-resistant Streptococcus pneumoniae: implications for understanding the role of the ribosome. Antimicrob. Agents Chemother..

[45] Wang J., Zhu Y., Guo J. (2022). Mechanisms of fluoroquinolone resistance in clinical isolates of Escherichia coli: a review. Front Microbiol.

[46] Dhand A., Mangal M., Kaur J. (2021). Vancomycin resistance in Enterococcus faecium: mechanisms and management. Infect. Drug Resist.

[47] Strahl H., Errington J. (2020). The cell wall of Gram-positive bacteria: a new perspective on an old topic. Nat. Rev. Microbiol.

[48] Da Costa J., Rojas A., Peirano G. (2022). Alteration of antibiotic target sites and its contribution to resistance in bacterial pathogens. Antibiot. (Basel).

[49] Piddock L.J. (2017). Multidrug-resistance efflux pumps? Not just a problem for antibiotics. Nat. Rev. Microbiol.

[50] Khoshnood S., Zali F., Ghaffari H.R. (2021). Enzymatic degradation and modification of antibiotics: mechanisms and implications for antimicrobial resistance. J. Antimicrob. Chemother..

[51] Chen Y., Chen J., Hu S. (2021). The role of horizontal gene transfer in the spread of antibiotic resistance in bacteria. Microbiol Res.

[52] Blair J.M., Webber M.A., Baylay A.J., Ogbolu D.O., Piddock L.J. (2015). Molecular mechanisms of antibiotic resistance. Nat. Rev. Microbiol.

[53] Munita J.M., Arias C.A. (2016). Mechanisms of antibiotic resistance. Microbiol Spectr..

[54] Diene S.M., Rolain J.M. (2014). Mechanisms of bacterial resistance: focus on efflux pumps, drug permeability, and target modification. Future Microbiol.

[55] Hofer U. (2022). Reduced permeability as a mechanism of antimicrobial resistance in Gram-negative bacteria. Nat. Rev. Microbiol.

[56] Delcour A.H. (2009). Outer membrane permeability and antibiotic resistance. Biochim Biophys. Acta.

[57] Pages J.M., James C.E., Winterhalter M. (2008). The porin and the permeating antibiotic: a selective diffusion barrier in Gram-negative bacteria. Nat. Rev. Microbiol.

[58] Li X.Z., Plésiat P., Nikaido H. (2015). The challenge of efflux-mediated antibiotic resistance in Gram-negative bacteria. Clin. Microbiol Rev..

[59] Blair J.M., Webber M.A., Baylay A.J., Ogbolu D.O., Piddock L.J. (2015). Molecular mechanisms of antibiotic resistance. Nat. Rev. Microbiol.

[60] Lomovskaya O., Bostian K.A. (2006). Practical applications and feasibility of efflux pump inhibitors in the clinic—a vision for applied use. Biochem Pharm..

[61] Delcour A.H. (2009). Outer membrane permeability and antibiotic resistance. Biochim Biophys. Acta.

[62] Stewart P.S. (2002). Mechanisms of antibiotic resistance in bacterial biofilms. Int J. Med Microbiol.

[63] Costerton J.W., Stewart P.S., Greenberg E.P. (1999). Bacterial biofilms: a common cause of persistent infections. Science.

[64] Partridge S.R., Kwong S.M., Firth N., Jensen S.O. (2018). Mobile genetic elements associated with antimicrobial resistance. Clin. Microbiol Rev..

[65] McInnes R.S., McCallum G.E., Lamberte L.E., van Schaik W. (2020). Horizontal transfer of antibiotic resistance genes in the human gut microbiome. Curr. Opin. Microbiol.

[66] Munita J.M., Arias C.A. (2016). Mechanisms of antibiotic resistance. Microbiol Spectr..

[67] Pesesky M.W., Hussain T., Wallace M., Patel S., Andleeb S., Burnham C.D. (2019). Evaluation of transfer and stability of antibiotic resistance in bacterial biofilms. Antimicrob. Agents Chemother..

[68] Perry J.A., Wright G.D. (2013). The antibiotic resistance "mobilome": searching for the link between environment and clinic. Front Microbiol.

[69] Culyba M.J., Mo C.Y., Kohli R.M. (2015). Targets for combating the evolution of acquired antibiotic resistance. Biochemistry.

[70] von Wintersdorff C.J., Penders J., van Niekerk J.M., Mills N.D., Majumder S., van Alphen L.B. (2016). Dissemination of antimicrobial resistance in microbial ecosystems through horizontal gene transfer. Front Microbiol.

[71] Frost L.S., Leplae R., Summers A.O., Toussaint A. (2005). Mobile genetic elements: the agents of open source evolution. Nat. Rev. Microbiol.

[72] Davies J., Davies D. (2010). Origins and evolution of antibiotic resistance. Microbiol Mol. Biol. Rev..

[73] Domingues S., Harms K., Fricke W.F., Johnsen P.J., da Silva G.J., Nielsen K.M. (2012). Natural transformation facilitates transfer of transposons, integrons and gene cassettes between bacterial species. PLoS Pathog..

[74] Li X.Z., Elkins C.A., Zgurskaya H.I. (2016).

[75] Singh P., Thakur M., Goel G. (2020). Investigating the role of bacteriophages in tackling antimicrobial resistance through bacterial genome editing. Front Microbiol.

[76] Tazzyman S.J., Bonhoeffer S. (2014). Why there are no essential genes on plasmids. Mol. Biol. Evol..

[77] Ghaly T.M., Geoghegan J.L., Tetu S.G., Gillings M.R. (2020). The effect of antibiotic exposure in a human impacted environment on the prevalence of integron-mediated antimicrobial resistance. Curr. Res Micro Sci..

[78] Nicolas-Chanoine M.H., Bertrand X., Madec J.Y. (2014). Escherichia coli ST131, an intriguing clonal group. Clin. Microbiol Rev..

[79] Blair J.M., Webber M.A., Baylay A.J., Ogbolu D.O., Piddock L.J. (2015). Molecular mechanisms of antibiotic resistance. Nat. Rev. Microbiol.

[80] Lerminiaux N.A., Cameron A.D. (2019). Horizontal transfer of antibiotic resistance genes in clinical environments. Can. J. Microbiol.

[81] Poirel L., Madec J.Y., Lupo A., Schink A.K., Kieffer N., Nordmann P. (2018). Antimicrobial resistance in Escherichia coli. Microbiol Spectr..

[82] Baltrus D.A. (2013). Exploring the costs of horizontal gene transfer. Trends Ecol. Evol..

[83] San Millan A. (2018). Evolution of plasmid-mediated antibiotic resistance in the clinical context. Trends Microbiol.

[84] Palmer K.L., Kos V.N., Gilmore M.S. (2010). Horizontal gene transfer and the genomics of enterococcal antibiotic resistance. Curr. Opin. Microbiol.

[85] Zhang L., Kinkel L.L., Newton G., Zhang B., Schroth M.N. (2014). Transfer of antibiotic resistance to other bacteria in soil. J. Environ. Sci. Technol..

[86] McArthur A.G., Wright G.D., Lengeler J.W., Drews G., Schlegel H.G. (2004). Biology of the prokaryotes.

[87] Furuya E.Y., Lowy F.D. (2006). Antimicrobial-resistant bacteria in the community setting. Nat. Rev. Microbiol.

[88] De la Cruz F., Davies J. (2000). Horizontal gene transfer and the origin of species: lessons from bacteria. Trends Microbiol.

[89] Van Hoek A.H., Mevius D., Guerra B., Mullany P., Roberts A.P., Aarts H.J. (2011). Acquired antibiotic resistance genes: an overview. Front Microbiol.

[90] Thomas C.M., Nielsen K.M. (2005). Mechanisms of, and barriers to, horizontal gene transfer between bacteria. Nat. Rev. Microbiol.

[91] Carattoli A. (2013). Plasmids and the spread of resistance. Int J. Med Microbiol.

[92] Gyles C.L., Boerlin P. (2014). Horizontally transferred genetic elements and their role in pathogenesis of bacterial disease. Vet. Pathol..

[93] Aminov R.I. (2011). Horizontal gene exchange in environmental microbiota. Front Microbiol.

[94] De Gelder L., Ponciano J.M., Joyce P., Top E.M. (2007). Stability of a promiscuous plasmid in different hosts: no guarantee for a long-term relationship. Microbiology.

[95] Zankari E., Hasman H., Cosentino S., Vestergaard M., Rasmussen S., Lund O. (2012). Identification of acquired antimicrobial resistance genes. J. Antimicrob. Chemother..

[96] Hooban B., Joyce A., Fitzhenry K., Chique C., Rogers L., Crispie F. (2020). The role of horizontal gene transfer in the evolution of the human gut microbiome. Genome Biol..

[97] Berendonk T.U., Manaia C.M., Merlin C., Fatta-Kassinos D., Cytryn E., Walsh F. (2015). Tackling antibiotic resistance: the environmental framework. Nat. Rev. Microbiol.

[98] Nguyen T.Q., Choudhary A., Heo B.E., Jeon S., Moon C., Park Y., Jang J. (2023). CRISPR Interference-Based Inhibition of MAB_0055c Expression Alters Drug Sensitivity in Mycobacterium abscessus. Microbiol. Spectr..

[99] Thakore P.I., Gemberling M.P., Nelson C.E., Oliver M.L., Gersbach C.A., Rouse D.C., Kwon J.B. (2018). RNA-guided transcriptional silencing in vivo with S. aureus CRISPR-Cas9 repressors. Nat. Commun..

[100] Luo M.L., Leenay R.T., Beisel C.L., Mullis A.S. (2014). Repurposing endogenous type I CRISPR-Cas systems for programmable gene repression. Nucleic Acids Res..

[101] Chen F., Chen F., Wang C., Wang Z., Wang Z., Xu L., White J.C., Xing B., Tao M., Wang Z., Tao M., Du H., Wang C., Xu L., Du H. (2024). Nitrogen-Doped Carbon Dots Facilitate CRISPR/Cas for Reducing Antibiotic Resistance Genes in the Environment. J. Agric. Food Chem..

[102] Kuroda M., Ohta T., Hayashi H., Wada A., Takeuchi F., Mori H. (2001). Whole genome sequencing of methicillin-resistant Staphylococcus aureus. Lancet.

[103] Sorensen S.J., Bailey M., Hansen L.H., Kroer N., Wuertz S. (2005). Studying plasmid horizontal transfer in situ: a critical review. Nat. Rev. Microbiol.

[104] Stokes H.W., Gillings M.R. (2011). Gene flow, mobile genetic elements and the recruitment of antibiotic resistance genes into Gram-negative pathogens. FEMS Microbiol Rev..

